# Neglected tropical diseases

**Published:** 2013

**Authors:** David Molyneux

**Affiliations:** Emeritus Professor: Centre for Neglected Tropical Diseases, Liverpool School of Tropical Medicine, Liverpool, UK.

**Figure:**
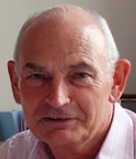
David Molyneux

## What are the neglected tropical diseases?

Seventeen neglected tropical diseases (NTDs) have been identified by the World Health Organization (WHO). It is estimated that over 1 billion people are infected with NTDs, with a further 1 billion at risk. The majority of NTDs occur in the tropics and sub-tropics and have particular characteristics in common:

- They afflict the poorest people – those without access to the safe water, sanitation, and basic health services required in order to protect themselves against infection by bacteria, viruses and other pathogens. High-income groups are rarely affected.
- Many are chronic, slowly developing conditions that become progressively worse if undetected and untreated. The damage they cause can be irreversible.
- They can cause severe pain and life-long disabilities, with long-term consequences for the person and also for family members who have to care for the person.
- People with NTDs are often stigmatised and excluded from society, and this can affect their mental health.

The individual diseases are very different, and one person can be affected by more than one disease at the same time.

**Figure:**
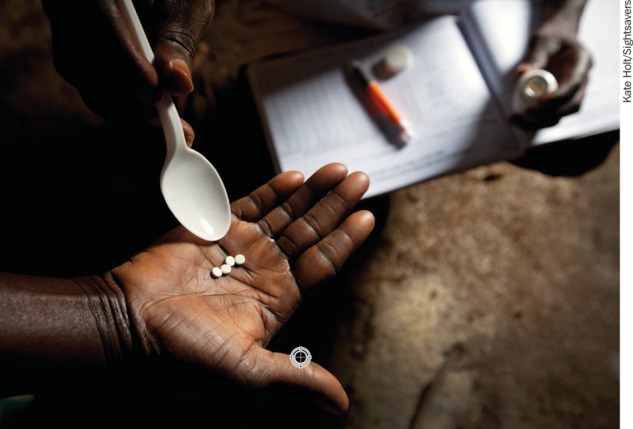
Community distribution of ivermectin for onchocerciasis. NIGERIA

The infectious agents responsible include:

- viruses (rabies and dengue)
- bacteria (leprosy, yaws, trachoma and Buruli ulcer)
- protozoa (leishmaniasis and trypanosomiasis)
- helminth parasites (schistosomiasis, lymphatic filariasis, onchocerciasis, intestinal worms and Guinea worm).

Transmission is equally diverse and can take place via:

- flies, fomites (e.g. skin cells, hair, clothing or bedding) and fingers (trachoma)
- mosquitoes (dengue fever and filariasis)
- tsetse flies (sleeping sickness)
- sandflies (leishmaniasis)
- blackflies (onchocerciasis)
- snails, which release infective larvae into water to penetrate human skin (e.g schistosomiasis)
- the faeco-oral route (e.g. soil-transmitted helminths-see page 29) or via food products.

NTDs can cause blindness (onchocerciasis and trachoma), deformity and disablement, disfigurement, cancers, and neurological problems.

**ABOUT THIS ISSUE**

**Figure:**
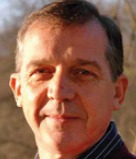
Allen Foster

Co-director: International Centre for Eye Health, London, UK.

In 1988, Merck in the USA made Mectizan available at no cost to communities with onchocerciasis infection. The commitment was ‘as much as is needed for as long as it is needed.^1^

This game-changing donation heralded the development of a new global partnership in health between the pharmaceutical industry, UN agencies, national ministries of health, non-governmental organisations and communities at risk – sectors of society which normally do not work together. Although they have different structures, driving forces, and skills, they agreed to break down the barriers that usually make them work in separate silos and to come together with a common vision to control and eliminate the specific diseases affecting neglected people. Two of these diseases – onchocerciasis and trachoma – cause blindness. Thanks to these ongoing donations, the challenge with neglected tropical diseases today is not so much to discover a treatment but rather to reach the very remote communities with an integrated, effective and sustainable programme of disease control.

This edition of the *Journal* aims to inform our readers about the neglected tropical diseases, the communities affected, and the available control measures. Emphasis is placed on integration and learning from each other to make the programmes more effective.

The biological diversity of NTDs means that the control or elimination strategies also are very diverse.

Several NTDs can be controlled by drug treatment (preventive chemotherapy), on a country or community scale, via mass drug administration programmes. Other NTDs require different approaches and strategies for control or elimination, including specialised drugs and/orvector control (limiting or eradicating insects-e.g. flies and bugs-that transmit the pathogens).

Despite the diversity of the strategies, however, there are good opportunities for comprehensive NTD elimination and control programmes.

## The social and economic impact of NTDs

NTDs are a result of poverty; they also contribute to further poverty in those people affected. Indeed, the prevalence of some NTDs has been suggested as an indicator of poverty.^1^, ^2^ They also have a wide social and economic impact:

- the loss of ability to undertake traditional farming practices, critical for survival in rural environments
- the loss of ability to play an economic and social role within the family and community
- the cost of inappropriate treatment (for example, traditional healers), which enhances the cycle of poor health
  and poverty
- the loss of educational opportunities, as children must act as caregivers for their parents, creating a generation of people with little or no education • poor mental health of the patient and the caregiver, particularly chronic depression.

The impact of NTDs on the unpaid work provided by women in the community is more difficult to measure. When women are ill, they are less able to do work such as growing vegetables, fetching water and fuel, providing care for older people and children, and ensuring that family members wash their hands or wear shoes -which reduces the transmission of NTDs. Women tend to have poorer access to health care than men and are also disproportionately affected by some NTDs, such as trachoma.^3^

## Why are NTDs receiving increased international recognition?

Over the last decade, NTDs have received increased recognition. This was made possible thanks to the establishment of NTDs as a ‘brand’ in global health.

It was difficult to focus the world's attention on 17 very different diseases requiring a range of different interventions. By recognising what these diseases had in common, and grouping them together under the NTD ‘brand’, however, it became possible to construct compelling arguments for action at the international level. These arguments were supported by good evidence: that addressing NTDs is cost effective in terms of economic rates of return on investment of health dollars, leading to ‘more health, for more people, for fewer dollars.’^4^ Further, the relationship between NTDs and social, equity/equality and development issues means they fall within the mandate of development agencies, therefore meriting both technical and financial support.

**Figure:**
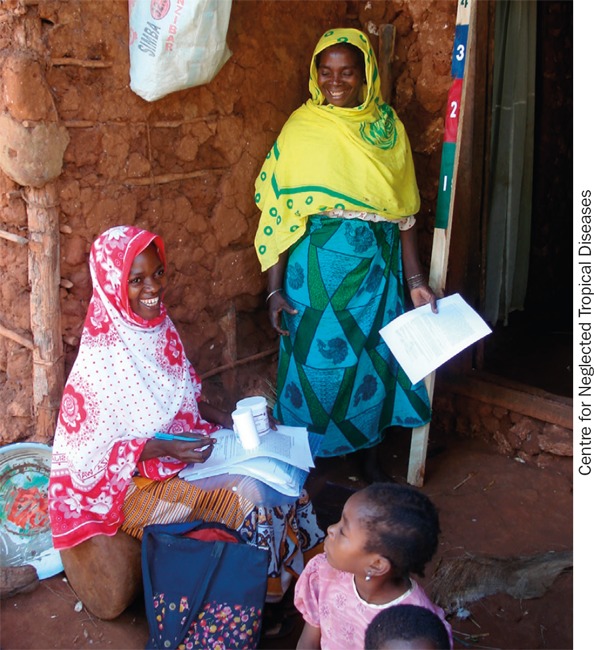
Treatment is brought to every home in the village. ZANZIBAR

## What is being done to control NTDs?

The drugs needed to treat NTDs are now included on the WHO ‘Essential Medicines’ list, and pharmaceutical companies are making them freely available to the populations in need through donation programmes.

These programmes, together with increased country commitment to the control of NTDs and novel approaches to drug distribution (e.g. through community-directed interventions or school health programmes) have made it possible to address some NTDs (trachoma, onchocerciasis, lymphatic filariasis, soil-transmitted helminths, and schistosomiasis) on a massive scale, in what have become known as mass drug administration (MDA) programmes.

Community-directed treatment has been developed and promoted as a recommended way of providing mass drug administration. Communities take responsibility for the collection, delivery, and reporting of drug use. This is an effective approach: annual treatment records for onchocerciasis suggest that some 70% of the ivermectin approved for use is administered to those who need it within a 12-month period.

Mass drug administration programmes bring multiple benefits, including:

- direct impact on individual health, even beyond the target infection
- improved community engagement in health programmes
- better access to health care for populations that had little or no access
- an improved drug supply chain
- enhanced data management, monitoring, evaluation, surveillance and reporting systems.

## What more needs to be done?

The majority of countries in Africa have completed their NTD master plans. Resources must be allocated to these plans and countries need to commit to the World Health Assembly (WHA) Resolution^5^ and the WHO Road Map.^6^

The second WHO Report on NTDs, ‘Sustaining the Momentum’^7^, was published recently and identified progress since the first report.^8^ It also identifies the challenges in the way of achieving the disease-specific goals.

There is a need for rapid up-scaling in some of the most populous countries to reach the WHO targets and timelines for control/elimination. To address this, NTD partners need to:

- engage in advocacy in their country complete mapping
- enhance human resource capacity in order to deliver integrated treatments within the health system
- address the backlog of surgery for some diseases (in particular, trachoma and lymphatic filariasis)
  **Table 1.** The 17 neglected tropical diseases, as defined by the World Health Organization

**Buruli ulcer**(*Mycobacterium ulcerans* infection)
**Chagas disease**
**Dengue/severe dengue**
**Dracunculiasis**(Guinea-worm disease)
**Food-borne trematodiases and fascioliasis** (liver flukes)
**Human African trypanosomiasis**(sleeping sickness)
**Human echinococcosis**(hydatid disease)
**Leishmaniasis**
**Leprosy**
**Lymphatic filariasis**
**Onchocerciasis**(river blindness)
**Rabies**
**Schistosomiasis** (bilharzia)
**Soil-transmitted helminthiases**
**Taeniasis/cysticercosis**(tapeworms)
**Trachoma**
**Yaws** (endemic treponematoses)
- implement a new strategy in areas where *Loa loa* (tropical eye worm) is co-endemic with lymphatic filariasis. This is due to the problems of severe reactions to ivermectin when people have high parasite loads of *Loa loa.*

**Action on neglected tropical diseases at the global level**

- On 27th May 2013, the WHA passed a resolution on all 17 neglected tropical diseases (Resolution WHA 66.20).^5^
- NTDs have been included in the post-2015 Development Agenda.^9^ Goal 4e states: ‘Reduce the burden of disease from HIV/AIDS, tuberculosis, malaria, neglected tropical diseases, and priority-non communicable diseases.’
- In early 2012, the London Declaration^10^ was endorsed by 77 companies and organisations. The declaration included an increased commitment to drug donations and product development research to support the World Health Organization (WHO) Road Map^5^ towards the specific 2012-2020 NTD targets.
- USAID and the UK Department for International Development have increased their support for the elimination of NTDs, and the Bill & Melinda Gates Foundation has committed significant research funds to address operational and product-oriented research for NTDs.

Globally, the investment required for the delivery of donated drugs is estimated at around US $0.50 per person treated, per year. Included in this ‘unit cost’ is the cost of training, social mobilisation, evaluation and monitoring, and surveillance, all of which are needed for mass drug administration programmes to be effective. The unit cost is estimated to be even lower in some settings: around US $0.10-0.20 per person treated, per year. Even in the poorest countries, this represents just a small fraction of the national per capita health expenditure.

## Conclusion

Programmes to eliminate and control NTDs address issues of equity (equal access to health care) and are interventions that directly benefit the poor. The drug treatments are effective and broadly safe when correct policies are followed (see page 26).

Mass drug administration programmes that reduce morbidity, mortality and transmission – leading to elimination of some of the world's most distressing diseases -should be regarded as akin to global immunisation when viewed from a strategic perspective. They have proved that it is possible to deliver free drugs to the poorest in need at unit costs that even some of the poorest countries can afford, and have already afforded. We must call for this successful intervention to be made available to everyone who needs treatment. If this relatively easy type of intervention – free drugs, no need for a cold chain – cannot be replicated and scaled up to reach everyone who needs treatment, worldwide, there is little hope that we can make a significant impact in other priority areas, such as maternal and child health, or vaccinations.

The NTD community has been successful in achieving a paradigm shift in the global health community's thinking about these diseases, as exemplified in a WHA Resolution and their inclusion in the a post-2015 Health Goal (see panel, page 23). What were hitherto unpronounceable conditions of poor people, and which did not concern high-income countries, are now high on the global health agenda. Consciousness has been raised but there remain many challenges, both technical and operational.

A higher level of commitment is needed from the endemic countries, additional donors, non-governmental organisations, and charities. NTD partnerships recognise that they must face the following challenges: **communicating** the need for **country commitment** to enhance geographic and therapeutic **coverage** and improve **compliance**, and achieving this by prioritising **capacity strengthening** from the **centre** to the **communities.**
